# High-Zinc Supplementation of Weaned Piglets Affects Frequencies of Virulence and Bacteriocin Associated Genes Among Intestinal *Escherichia coli* Populations

**DOI:** 10.3389/fvets.2020.614513

**Published:** 2020-12-16

**Authors:** Vanessa C. Johanns, Lennard Epping, Torsten Semmler, Fereshteh Ghazisaeedi, Antina Lübke-Becker, Yvonne Pfeifer, Inga Eichhorn, Roswitha Merle, Astrid Bethe, Birgit Walther, Lothar H. Wieler

**Affiliations:** ^1^Advanced Light and Electron Microscopy (ZBS-4), Robert Koch Institute, Berlin, Germany; ^2^Microbial Genomics (NG1), Robert Koch Institute, Berlin, Germany; ^3^Center for Infection Medicine, Institute of Microbiology and Epizootics, Freie Universität Berlin, Berlin, Germany; ^4^Nosocomial Pathogens and Antibiotic Resistance (FG13), Robert Koch Institute, Wernigerode, Germany; ^5^Institute for Veterinary Epidemiology and Biostatistics, Freie Universität Berlin, Berlin, Germany; ^6^Robert Koch Institute, Berlin, Germany

**Keywords:** *E. coli*, zinc, pig, virulence associated genes, bacteriocins, gut

## Abstract

To prevent economic losses due to post-weaning diarrhea (PWD) in industrial pig production, zinc (Zn) feed additives have been widely used, especially since awareness has risen that the regular application of antibiotics promotes buildup of antimicrobial resistance in both commensal and pathogenic bacteria. In a previous study on 179 *Escherichia coli* collected from piglets sacrificed at the end of a Zn feeding trial, including isolates obtained from animals of a high-zinc fed group (HZG) and a corresponding control group (CG), we found that the isolate collection exhibited three different levels of tolerance toward zinc, i.e., the minimal inhibitory concentration (MIC) detected was 128, followed by 256 and 512 μg/ml ZnCl_2._ We further provided evidence that enhanced zinc tolerance in porcine intestinal *E. coli* populations is clearly linked to excessive zinc feeding. Here we provide insights about the genomic make-up and phylogenetic background of these 179 *E. coli* genomes. Bayesian analysis of the population structure (BAPS) revealed a lack of association between the actual zinc tolerance level and a particular phylogenetic *E. coli* cluster or even branch for both, isolates belonging to the HZG and CG. In addition, detection rates for genes and operons associated with virulence (VAG) and bacteriocins (BAG) were lower in isolates originating from the HZG (41 vs. 65% and 22 vs. 35%, *p* < 0.001 and *p* = 0.002, resp.). Strikingly, *E. coli* harboring genes defining distinct pathotypes associated with intestinal disease, i.e., enterotoxigenic, enteropathogenic, and Shiga toxin-producing *E. coli* (ETEC, EPEC, and STEC) constituted 1% of the isolates belonging to the HZG but 14% of those from the CG. Notably, these pathotypes were positively associated with enhanced zinc tolerance (512 μg/ml ZnCl_2_ MIC, *p* < 0.001). Taken together, zinc excess seems to influence carriage rates of VAGs and BAGs in porcine intestinal *E. coli* populations, and high-zinc feeding is negatively correlated with enteral pathotype occurrences, which might explain earlier observations concerning the relative increase of *Enterobacterales* considering the overall intestinal microbiota of piglets during zinc feeding trials while PWD rates have decreased.

## Introduction

The gastrointestinal microbiota of pigs is a large, multifaceted and complex microbial community which has been estimated to comprise of 10^10^-10^11^ bacteria per gram of gut content (1). Different stress factors are capable of altering the healthy gut microbiome of a piglet, e.g., weaning from the sow, which is among the most stressful events in a piglet's life (2): The prompt change in diet, social and environmental life conditions affects the gut microbiota in composition and structure and prevalently enhances vulnerability for onset of enteral post-weaning infections (3). Considering feed additives, different products are marketed to assist in boosting the pigs' immune system, regulate gut microbiota, and reduce negative impacts of weaning and other environmental challenges, including pharmacological levels of zinc and copper (4). However, a recent review concluded that it is not possible to recommend a specific additive that will have positive effects in all diets, since the effects strongly depend on the health status of the animals (4). Therefore, maintaining a physiological/healthy gut microbiota composition and equilibrium during and after weaning is important to prevent attachment, proliferation and spread of pathogenic microorganisms, especially enterotoxigenic *Escherichia coli*, which is a frequent cause of post-weaning diarrhea (PWD) in piglets (5).

*E. coli* causing intestinal diseases usually express virulence factors which induce and/or support an inflammation of the gut often leading to diarrhea, while the number of intestinal commensals decreases (6). Complex mechanisms of interaction between commensal and pathogenic bacteria have evolved within the gut, including competition for nutrients, shielding from the activated enteral immune response and induction of competitor-eliminating bacteriocin production (7). These bacteriocins are polypeptide toxins comprising colicins and microcins which exhibit a broad range of different cytotoxic mechanisms (8–10). Both colicins and microcins are capable of killing a narrow spectrum of competing *Enterobacterales*, including other *E. coli* lineages (9). Porcine pathogenic *E. coli* have been shown to produce predominantly colicins, especially colicin BM and Ib (11).

To prevent or mend PWD which is most commonly caused by enterotoxigenic *E. coli* (12), high-level dietary zinc oxide supplementation is used in the pig production sector in different parts of the world (5, 13, 14). Although the particular effects of zinc on the enteral microbiota are not fully understood yet, piglets fed with high-zinc supplemented diets clearly showed changes with respect to the overall composition and abundance of distinct gut-associated bacteria (15–17), which are probably associated with bacteriocin-producing bacteria as well. We therefore hypothesized that the occurrence of genes encoding bacteriocins might have an impact on the composition of the *E. coli* population in zinc-fed piglets.

The manifestation of clinical symptoms and pathology of *E. coli-*induced enteral diseases is closely associated with the occurrence of certain virulence associated genes (VAGs) (18, 19). Mainly based on the presence of distinct VAGs but also additional, especially phenotypical characteristics, diarrheagenic *E. coli* of importance for the pig production sector are often classified as enterotoxigenic *E. coli* (ETEC). Furthermore, enteropathogenic *E. coli* (EPEC), Shiga toxin-producing *E. coli* (STEC), enteroaggregative *E. coli* (EAEC), enteroinvasive *E. coli* (EIEC) and diffusely adherent *E. coli* (DAEC) have been reported as a probable cause of diarrhea in pigs as well (5, 19–21).

While we have recently shown that high-zinc supplemented diets foster accumulation of *E. coli* associated with increased zinc-tolerance in weaned piglets (22), the effects of that particular feed additive on the occurrence of VAGs and bacteriocin associated genes (BAGs) among the intestinal *E. coli* population of these piglets were not investigated so far. In the current study, we analyzed the phylogenetic relationship and make up of a broad collection of *E. coli* obtained from a former piglet zinc-feeding trial together with strains isolated from pigs suffering from clinical disease in order to investigate the presence of a putative correlation between feeding group and the zinc tolerance level of the isolates with the occurrence of VAGs and/or BAGs.

## Materials and Methods

### Animal Trial and Bacterial Isolates

The representative set of *E. coli* isolates characterized here was selected based on a previous feeding trial (23) carried out in accordance with the principles of the Basel Declaration following the institutional and national guidelines for the care and use of animals. The protocol was approved by the local state office of occupational health and technical safety “Landesamt für Gesundheit und Soziales, Berlin” (LaGeSo Reg. Nr. 0296/13) as described before (23).

Briefly, 32 landrace piglets of regular commercial origin weaned at day 25 ± 1 were separated into two groups for 4 weeks: the first group of piglets, designated here as the high-zinc group (HZG) was fed with a diet supplemented with a comparatively high amount of zinc oxide (2,103 mg zinc/kg diet), while the second group served as the control group. This control group (CG) received a common piglet diet containing a concentration of zinc oxide (72 mg zinc/kg diet) sufficient to meet the nutritional requirements to avoid trace metal malnutrition (23). The trial started with 32 piglets, which were sacrificed mid-trial (38 ± 2 days of age, *n* = 16, 8 per group) and at the trial's end (52 ± 2 days of age, *n* = 16, 8 per group). No symptoms of disease were observed within the two feeding groups during the entire trial. Here we focus on *E. coli* obtained from three different sampling sites (feces, digesta and mucosa obtained from the colon ascendens) of those piglets sacrificed at the end (52 ± 2 days of age; *n* = 16) of the feeding trial. In total, 179 *E. coli* were collected as a stratified random sample (HZG = 99, CG = 80), with an average number of 11 *E. coli* isolates investigated per piglet (all three sampling sites). These isolates were previously studied with regard to their respective zinc tolerance levels and susceptibility patterns for antibiotics and biocides (22).

To compare, we chose six additional porcine strains isolated from clinically ill piglets [diarrhea (*n* = 4), edema disease (*n* = 2)] available in the strain collection of the Institute of Microbiology and Epizootics (IMT) as representatives for non-commensal isolates and a high-zinc tolerant isolate from a clinical human case (RKI6122) (24).

### Whole Genome Sequencing, Phylogenetic Analysis, and Screening for Virulence Associated- (VAGs) and Bacteriocin Associated Genes (BAGs)

*E. coli* were sequenced using Illumina MiSeq® 300 bp paired-end whole genome sequencing (WGS) with an obtained coverage of >90x. The Illumina short reads were hybrid assembled using unicycler v4.4 (25). Adapter-trimmed reads were used for *de novo* assembly into contiguous sequences (contigs) and subsequently into scaffolds using SPAdes v3.11. All draft genomes were annotated using Prokka v1.14.5 (26). Illumina raw read data sequenced for this study is available at NCBI under Bioproject ID PRJNA552271. The determination of the maximum common genome (MCG) (27) alignment was done comprising those genes present in all 179 genomes. To obtain this, we clustered the coding sequences based on the parameters sequence similarity (min. 90%) and coverage (min. 90%) and defined the genes that were present in each genome, fulfilling the threshold parameters as MCG. This resulted in 2,804 orthologous genes that we used for the comparisons. We extracted the allelic variants of these genes from all genomes by a BLAST-based approach, aligned them individually for each gene and concatenated them, which result in an alignment of 2.762 Mbp for these 179 strains. This alignment was used to generate a phylogenetic tree with RAxML v 8.2.10 (28) using a General Time Reversible model and gamma correction for among site rate variation. Bayesian analysis of population structure (BAPS) (29) was applied to identify genetically distinct linages based on the constructed phylogeny.

An in-house BLAST-pipeline with the general gene identity threshold of 95 and 90% minimum coverage was used to identify 25 VAGs selected because of their (putative) importance in intestinal pathogenicity (Table 1). Further genotype characterization included the determination of multilocus sequence type (ST) and sequence type complex (STC) using MLST 2.0 (35), and serotype prediction using SerotypeFinder 2.0 (36).

**Table 1:** Occurrence and distribution of VAGs associated with different types of intestinal pathogenic *E. coli* of relevance for pigs.

			**ZnCl**_****2****_ **MIC (μg/ml)**						**Feeding group**			
			**128**		**256**		**512**		**HZG**		**CG**	
			***n***	**%**	***n***	**%**	***n***	**%**	***n***	**%**	***n***	**%**
**Gene**	**Protein**	**Associated pathotype**	**7**	**100**	**136**	**100**	**36**	**100**	**99**	**100**	**80**	**100**
*aid*A	adhesin involved in diffuse adherence	DAEC/ETEC	3	43	15	11	3	8	4	4	17	21
*aah*	AT adhesin heptosyltransferase	DAEC/ETEC	3	43	15	11	3	8	4	4	17	21
*bfp*A	type IV bundle-forming pili	EPEC										
*eae*	Intimin	STEC/EPEC			12	9			1	1	11	14
*fae*G	F4 fimbrial adhesin	ETEC										
*fas*A	F6 fimbrial adhesin	ETEC										
*fanC*	F5 fimbrial adhesin	ETEC										
*fed*A	F18 fimbrial adhesin	ETEC										
*f*41	F41 fimbrial adhesin	ETEC										
*iha*	novel non-hemagglutinin adhesin	STEC			12	9	19	53	20	20	11	14
*pic*	serine protease autotransporter	EAEC			18	13	4	11	9	9	13	16
*paa*	porcine AE associated protein	STEC/EPEC			10	7			1	1	9	11
*saa*	STEC autoagglutinating adhesin	STEC										
*cdt*	cytolethal distending toxin	EPEC					2	6	2	2		
*ast*A/ *east*1	enterohemolysin	EAEC			31	23	4	11	9	9	26	33
*ehx*A	enterohemolysin	EPEC			10	7			1	1	9	11
*efa*1/ *lif*A	lymphostatin	EPEC			10	7			1	1	9	11
*elt*AB	heat-labile (LT) enterotoxin subunit A and B	ETEC	2	29			8	22	6	6	4	5
*est*A	heat-stable enterotoxin STa	ETEC			4	3	7	19	7	7	4	5
*est*B	heat-stable enterotoxin STb	ETEC			12	9	2	6	3	3	11	14
*set*1AB	Shigella enterotoxin 1, ShET1	EAEC			2	2	1	3	2	2	1	1
*stx*_1_	Shiga toxin 1 (subunit A and B)	STEC			2	2					2	3
*stx*_2_	Shiga toxin 2 (subunit A and B)	STEC			14	10					14	18

*All 179 porcine E. coli from both feeding group (HZG and CG) were screened with respect to the presence or absence of 13 different adhesion and 11 different toxin encoding genes associated with intestinal E. coli pathotypes. References for the major diagnostic markers are provided by Ref. (18–20, 30–34)*.

*n, number of isolates; HZG, high zinc fed group; CG, control group; VAG, virulence associated genes; DAEC, diffuse adherent E. coli, ETEC, enterotoxigenic E. coli; STEC, Shiga toxin-producing E. coli; EPEC, enteropathogenic E. coli, EAEC, enteroaggregative E. coli*.

The screening procedure for bacteriocin associated genes included colicin types depending on either a Tol-dependent translocation system (group A) such as colicin A, E1–E4, E6–E9, K, N, S4, U, Y or a Ton system (group B) including colicin B, Ia, Ib, E5, E7, 5, 10, G, H, Js, D, and M (37–41). An in-house BLAST-pipeline was set-up for each of the corresponding genes with the general identity threshold of 95 and 90% minimum gene coverage (Supplementary Figure 1) (9, 41).

Each gene encoding a bacteriocin (colicin, microcin) was further investigated with respect to its predicted amino acid (aa) sequence coverage and identity using corresponding reference/prototype aa sequences of the particular *E. coli* protein from NCBI (Supplementary Figure 1). An *in silico* comparative analysis was performed on these aa sequences using Geneious Prime (version 2019.0.04).

A detailed overview of the characteristics of all animal trial isolates and the strains included for comparative purposes are provided in Supplementary Tables 1, 2.

### Statistical Analysis

Data were analyzed using SPSS software version 25.0 (IBM, New York, NY, USA). *P*-values < 0.05 were considered statistically significant.

A mixed-model regression approach was used to test whether the feeding group and *E. coli* ZnCl_2_ MICs had an effect on the total number of different VAGs, with the individual pig as random factor. Since the dependent variable showed a Poisson distribution, Poisson regression and logarithmic link function were applied, with feeding group and ZnCl_2_ MICs (< vs. > = 512) included as factors into the model. Interactions were tested and removed when the *p*-value was > 0.05. Variance components analysis was used to determine the proportion of variance that accounted for differences between individual animals. In a second mixed logistic regression model, the individual effects of the above mentioned influence factors on the presence of major diagnostic markers was tested. Interactions were also tested as described above.

## Results

### Population Structure Analysis of Porcine *E. coli* of Different Feeding Groups and Zinc Tolerance Levels

Considering the overall phylogenetic diversity, we identified 2,804 orthologous genes representing the “maximum common genome” (MCG) of 186 genomic sequences representing isolates from both feeding groups (HZG = 99, CG = 80), six isolates previously collected from diagnostic samples of severely ill piglets (diarrhea, edema disease) and one further isolate of human origin [RKI6122 (24)] which exhibited a MIC value of 1,024 μg/ml ZnCl_2_.

**Figure 1:**
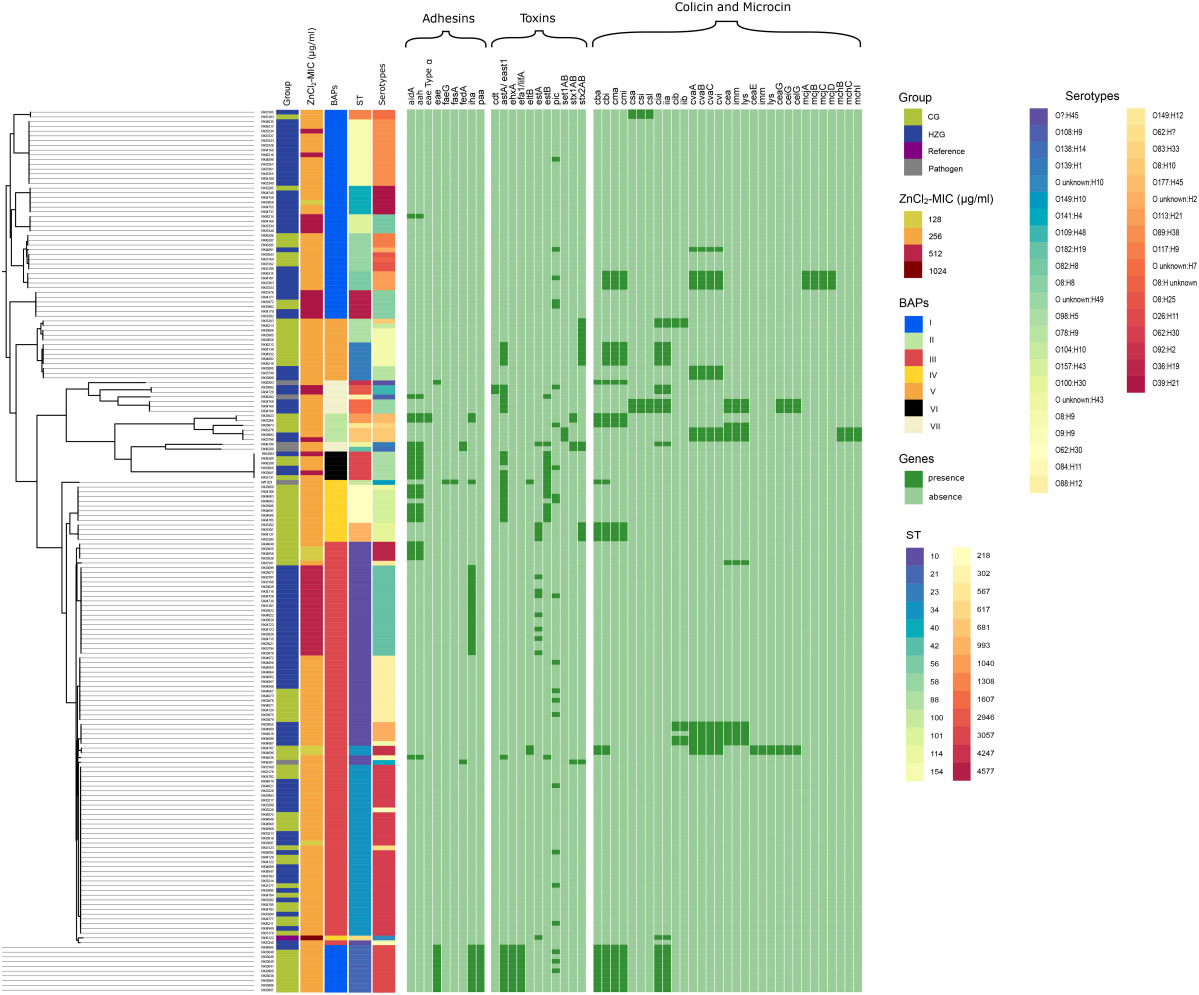
Maximum Common Genome Alignment of 179 commensal *E. coli* isolates, six porcine pathogenic *E. coli* and a high-zinc tolerant reference. Information about feeding group, ZnCl_2_ MIC values, BAPS (Bayesian analysis of the population structure (BAPS) clusters, sequence types, serotypes, presence (dark green) and absence (light green) of genes encoding adhesins, toxins, colicins, and microcins are presented from left to right.

The phylogeny was constructed from the MCG alignments of 186 genomes (Figure 1) and further investigated using Bayesian Analysis of Population Structure (BAPS). Seven distinct phylogenetic groups were identified, namely the BAPS cluster I-VII (Figure 1). BAPS cluster I comprised 54 isolates of both feeding groups (HZG: *n* = 35; CG: *n* = 19) distributed among eight STs, half of which containing strains with a ZnCl_2_ MIC of 256 μg/ml [ST21 (*n* = 10), ST56 (*n* = 4), ST58 (*n* = 8), and ST1308 (*n* = 2)]. ST101 (*n* = 4) and ST4577 (*n* = 6) isolates showed a ZnCl_2_ MIC of 512 μg/ml, including the only two CG-isolates with this MIC. ST154 (*n* = 14) isolates were associated with ZnCl_2_ MICs of either 256 (*n* = 12) or 512 μg/ml (*n* = 2), and ST40 (*n* = 6) isolates showed ZnCl_2_ MICs of 128 μg/ml (*n* = 1) and 256 μg/ml (*n* = 5).

Cluster II (*n* = 6; HZG: *n* = 2; CG: *n* = 4) contained five isolates associated with the 256 μg/ml ZnCl_2_ MIC including ST567 (*n* = 1), ST681 (*n* = 2), and ST1040 (*n* = 2). A third ST681 isolate of cluster II showed a 512 μg/ml ZnCl_2_ MIC.

With 84 isolates, BAPS cluster III formed the largest phylogenic group and consisted exclusively of isolates belonging to STC10 (HZG: *n* = 51; CG: *n* = 32; pathogenic isolate: *n* = 1). In more detail, ST10 (*n* = 45) isolates with distinct ZnCl_2_ MICs [128 μg/ml (*n* = 3), 256 mg/ml (*n* = 23), and 512 μg/ml (*n* = 19) ZnCl_2_], and ST34 (*n* = 38) isolates with either 128 μg/ml (*n* = 3) or 256 μg/ml (*n* = 35) ZnCl_2_ MICs, respectively, belonged to cluster III. In addition, one STEC isolate (IMT4632) isolated from a pig suffering from diarrhea (ST10; 256 μg/ml ZnCl_2_ MIC) clustered here, too.

Because of “long-branch attraction” artifacts (42), 12 CG isolates belonging to two different phylogenetic lineages (ST218, *n* = 8 and ST993, *n* = 4) with a ZnCl_2_ MIC value of 256 μg/ml were assigned to BAPS cluster IV. Also, two of the strains included for comparative reasons, i.e., one porcine ETEC-isolate (IMT203; ST100; 256 μg/ml ZnCl_2_ MIC) and the high-zinc tolerant human isolate (RKI6122; ST617; 1,024 μg/ml ZnCl_2_ MIC), clustered in BAPS IV, too.

Cluster V included 13 STC23-isolates (ST23, *n* = 8 [HZG: *n* = 3; CG: *n* = 5] and ST88, *n* = 5 [all CG]) associated with the 256 μg/ml ZnCl_2_ MIC. Six isolates of ST3057 (HZG: *n* = 3, CG: *n* = 3) were assigned to BAPS cluster VI (256 μg/ml ZnCl_2_ MIC, *n* = 4; 512 ZnCl_2_ MIC, *n* = 2).

Last, BAPS cluster VII consisted of ST1607 (*n* = 3) isolates associated with 256 μg/ml ZnCl_2_ MIC and ST2946 (*n* = 2) isolates associated with 512 μg/ml ZnCl_2_ MIC, all originating from animals of the HZG. Four of the pathogenic *E. coli* isolates included in our analysis (IMT19–ST42, STEC; IMT20–ST114, STEC; IMT6655–ST4247, ETEC; and IMT8071–ST302, EPEC; all associated with 256 μg/ml ZnCl_2_ MIC) clustered here, too (Supplementary Table 3).

In other words, the BAPS cluster analysis mirrored the predicted serotypes and STs, irrespective of the individual isolate's zinc tolerance level, clearly rejecting the idea of a directional association between a certain *E. coli* phylogenetic lineage and a particular zinc tolerance level or feeding group.

### Distribution of VAGs in *E. coli* Genomes of the HZG and the CG

To assess the virulence potential of the isolate collection, we screened all genomes for the presence of genes that have frequently been reported as being associated with porcine enteral diseases (Supplementary Table 1). As a result, 18 out of 24 targeted genes were identified in at least one of the screened genomes (Table 1), with the number of VAGs per isolate ranging from 0 VAGs (*n* = 86 isolates) to a maximum of 7 VAGs (*n* = 3). Interestingly, 93% of the isolates originating from the HZG but 72% of the CG isolates harbored none, one, or two VAG(s) only. Isolates harboring more than two VAGs constituted 7% of the HZG and 28% of the CG isolates, indicating an influence of high-zinc diets on the intestinal microbiota with respect to VAG-carrying *E. coli*. Regression analysis revealed that the feeding group (*p* < 0.001) as well as the respective ZnCl_2_ MIC (*p* < 0.001) were associated with the number of VAGs present in an isolate. For instance, isolates belonging to the CG had 4.4 times higher odds for harboring higher numbers of VAGs (95% confidence interval: 2.0–9.8) compared to *E. coli* from the HZG. However, the odds ratio to carry higher numbers of VAGs was 4.2 (95% confidence interval 2.4–7.4) for *E. coli* associated with the 512 μg/ml ZnCl_2_ MIC when compared to the lower MICs.

Since most of the VAGs frequently associated with enteral diseases in piglets are carried by highly mobile genetic elements (43–46), divergent distribution of VAGs even beyond ST/serotype boundaries might occur. While there was an obvious association between ST/serotypes and VAG pattern, we also noted variation, even between isolates of the same serotype obtained from one piglet (e.g., pig 1, ST21 O26:H11 or pig 4, ST10 O182:H19) (Supplementary Table 1, Figure 1).

Since all isolates were originally obtained from healthy piglets kept under the defined conditions of an animal experiment, the overall low detection rates for enterotoxin and adhesion encoding genes, especially for ETEC-associated fimbriae (0%), was not surprising. However, all genes encoding the 11 toxins of interest here were identified among the 179 *E. coli* (Table 1). Again, isolates belonging to the CG harbored more toxin encoding genes than those obtained from the HZG (56 vs. 18%).

Eight of the 179 isolates were positive for the genes encoding the heat-labile enterotoxin *elt*AB (HZG, *n* = 6; CG, *n* = 2). Twenty-five isolates carried either the heat-stable enterotoxin gene *est*A (HZG, *n* = 7; CG, *n* = 4) or *estB* (HZG, *n* = 3; CG, *n* = 11) (Table 1).

Shiga toxin encoding genes were completely absent in *E. coli* representing the HZG, while 16 isolates of the CG were positive (20%, *n* = 16: *stx*_1_ *n* = 2 and *stx*_2e_ *n* = 14). Genes encoding other toxins including *ast*A encoding the enteroaggregative *E. coli* heat-stable enterotoxin EAST-1 (HZG, *n* = 9; CG, *n* = 26), enterohemolysin-encoding *ehxA* (HZG, *n* = 1; CG *n* = 9) or *efa*-1/*lifA* (HZG, *n* = 1; CG, *n* = 9) were detected in genomes from isolates of both feeding groups (Table 1, Supplementary Table 1).

Considering additional factors involved in the onset of PWD in piglets (47), 21 isolates (HZG, *n* = 4; CG, *n* = 17) harbored genes encoding an adhesion-involved-in-diffuse-adherence (AIDA-I) autotransporter and the AIDA-associated heptosyltransferase (*aah*) required to fully activate AIDA-I (Table 1). While 14% (*n* = 11) of the CG-isolates were found positive for the *eae*- encoded intimin associated with attaching and effacing, only one (1%) HZG isolate (RKI4865, ST21) was *eae*-positive, too.

According to the VAG profile, the six clinical porcine isolates included comprised two enterotoxigenic *E. coli* (ETEC—*elt*AB- and/or *est*B-positive), two Shiga toxin-producing *E. coli* (STEC—*stx*_1−_ and *stx*_2_-positive), one ETEC/STEC-hybrid (*estA, estB, stx*_1_, *stx*_2_), and one enteropathogenic *E. coli* (aEPEC—*eae*-positive), respectively (Supplementary Table 2).

### Occurrence of Pathotypes Among *E. coli* Isolates With Different Zinc Tolerance Levels

The *E. coli* isolates obtained from piglets fed with a high-zinc supplemented diet showed generally a lower VAG frequency. Based on this finding, we further investigated whether a specific pathotype is associated with a particular zinc tolerance level considering the previously determined ZnCl_2_ MIC values of 128, 256, or 512 μg/ml (22, 24) or not.

Our results showed that major diagnostic markers defining an isolate as ETEC were associated with all three different zinc tolerance levels and both feeding groups (Table 2). Moreover, ETEC showed a broad heterogeneity with respect to their phylogenetic backgrounds. However, genes encoding for ETEC-associated fimbriae F4, F5, F6, F18, and F41 were not identified. VAG combinations (*eae, paa*) that define porcine atypical EPEC (aEPEC) expressed the 256 μg/ml ZnCl_2_ MIC only, and were predominately detected among CG isolates (one exception) (Table 2). Isolates showing STEC characteristics were associated with the 256 μg/ml ZnCl_2_ MIC and the CG only.

**Table 2:** Distribution of VAG profiles including major diagnostic markers and additional determinants associated with intestinal *E. coli* pathotypes.

		**ZnCl_**2**_ MIC**	**Feeding group**					
**Serotype**	**ST**	**μg/ml**	**HZG**	**CG**	**Pathotype**	**Major diagnostic marker**	**Additional virulence determinants identified**	**REF**
O92:H2	ST34	128	0	2	ETEC	*elt*AB		(19, 34, 48)
O26:H11	ST21	256	0	7	aEPEC	*eae*	*iha, paa, ehx*A, *efa*1/*lif*A	(49)
O26:H11	ST21	256	1	2	aEPEC	*eae*	*iha, paa, pic, ehx*A, *efa1*/*lif*A	(49)
O177:H45	ST1040	256	0	2	STEC	eae, stx1	*iha, aid*A, *aah*	(50)
O84:H11	ST10	256	0	1	ETEC	*est*B		(19, 34, 48)
O157:H43	ST218	256	0	5	ETEC	*est*B	*aid*A, *aah*	(19, 34, 48)
O157:H43	ST218	256	0	1	ETEC	*est*B	*aid*A, *aah, pic*	(19, 34, 48)
Ounknown:H43	ST218	256	0	1	ETEC	*est*B	*aid*A, *aah*	(19, 34, 48)
O100:H30	ST993	256	0	4	ETEC/STEC	*est*A, *stx*2e		(19, 34, 48)
O98:H5	ST3057	256	0	3	ETEC	*est*B	*aid*A, *aah*	(19, 34, 48)
O98:H5	ST3057	256	1	0	ETEC	*est*B	*aid*A, *aah, pic*	(19, 34, 48)
O8:H8	ST4577	512	4	2	ETEC	*elt*AB		(19, 34, 48)
O8:H9	ST23	256	0	5	STEC	*stx*2e		(50)
O8:H9	ST88	256	0	3	STEC	*stx*2e		(50)
O8:H10	ST88	256	0	1	STEC	*stx*2e		(50)
O104:H10	ST88	256	0	1	STEC	*stx*2e		(50)
O182:H19	ST10	512	7	0	ETEC	*est*A	*iha*	(19, 34, 48)
O98:H5	ST3057	512	2	0	ETEC	*est*B	*aid*A, *aah*	(19, 34, 48)
O109:H48	ST2946	512	1	0	ETEC	*elt*AB	*cdt, pic*	(19, 34, 48)
O109:H48	ST2946	512	1	0	ETEC	*elt*AB	*cdt*	(19, 34, 48)

*Screening results for 179 porcine E. coli with respect to the presence of major diagnostic markers defining an intestinal pathotype of importance for pigs. Sequence- and serotypes of isolates representing two different feeding groups (HZG, CG) and their respective ZnCl_2_ MICs are shown. References (REF) for the major diagnostic markers and additional virulence genes (19, 34, 48–50)*.

*ST, sequence type; HZG, high-zinc fed group; CG, control group; VAG, virulence associated genes; ETEC, enterotoxigenic E. coli; aEPEC atypical enteropathogenic E. coli, STEC, Shiga toxin-producing E. coli*.

The mixed logistic regression model showed that both, the ZnCl_2_ MIC and the feeding group were associated with the occurrence of major diagnostic markers defining an enteral pathotype in the present *E. coli* collection. The probability for harboring major diagnostic markers defining an enteral pathotype was lower in the HZG than in the CG [*p* = 0.002, odds ratio 47.6 (95%-confidence interval: 4.3–500)]. However, the ZnCl_2_ MIC 512 resulted in 58.6-fold higher odds for the presence of a major diagnostic marker (*p* < 0.001; 95% confidence interval 7.4–467.7). Nevertheless, 77% of the total variation was associated with variation between individual animals, indicating a greater similarity of *E. coli* obtained from one animal than between those of different pigs.

### Distribution of BAGs in *E. coli* Genomes of the HZG and the CG

Amongst others, to kill or at least suppress the growth of non-host specific bacteria is one of the most important functions of the host-microbiota, a challenge which is fostered, beyond others, by production of plasmid-borne colicins and/or microcins (37, 51). To answer the question about whether genes required for colicin or microcin production were diversely distributed in *E. coli* obtained from different feeding groups, we screened the genomes under investigation accordingly. A detailed overview of information on aa sequence with the respective reference sequences used here for BAG identification is provided in Supplementary Figure 1.

Overall, 28 of 80 (35%) *E. coli* obtained from piglets belonging to the CG and 22 of 99 (22%) from the HZG harbored at least one BAG (Table 3). The most frequently detected BAGs were operons encoding for colicin B (*cba*+*cbi*: HZG *n* = 1; CG *n* = 18; *cbi* only, HZG *n* = 4; CG *n* = 5) and colicin M (HZG *n* = 5, CG *n* = 21) which are commonly co-located on a Col plasmid [(52), 35]. In addition, the plasmid carrying the colicin Ia encoding operon had a lower occurrence among the HZG isolates (HZG *n* = 6 vs. CG *n* = 16). Contrarily, the plasmid carrying the operon encoding for microcin V, one of the few actively secreted microcins (53), was more frequently associated with HZG isolates (HZG *n* = 15 vs. CG *n* = 3).

**Table 3:** Occurrence and distribution of 31 BAGs among 179 porcine *E. coli*.

		**ZnCl**_****2****_ **MIC (μg/ml)**						**Feeding group**			
		**128**		**256**		**512**		**HZG**		**CG**	
		***n***	**%**	***n***	**%**	***n***	**%**	***n***	**%**	***n***	**%**
**Gene**	**Protein**	**7**	**100**	**136**	**100**	**36**	**100**	**99**	**100**	**80**	**100**
*cba*	Colicin B	2	29	17	13			1	1	18	23
*cbi*		2	29	26	19			5	5	23	29
*cma*	Colicin M			26	19			5	5	21	26
*cmi*				26	19			5	5	21	26
*csa*	Colicin S4			5	4			4	4	1	1
*csi*				5	4			4	4	1	1
*csl*				5	4			4	4	1	1
*cia*	Colicin Ia			20	15	2	6	6	6	16	20
*iia*				20	15	2	6	6	6	16	20
*cib*	Colicin Ib			6	4			4	4	2	3
*iib*				6	4			4	4	2	3
*cea*	Colicin E1			12	9	1	3	10	10	3	4
*imm*				12	9	1	3	10	10	3	4
*lys*				12	9	1	3	10	10	3	4
*cea*E	Colicin E5	2	29							2	3
*imm*		2	29							2	3
*lys2*		2	29							2	3
*cea*G	Colicin E7	2	29	3	2			3	3	2	3
*cei*G		2	29	3	2			3	3	2	3
*cel*G		2	29	3	2			3	3	2	3
*cva*A	Microcin V	2	29	15	11	1	3	15	15	3	4
*cva*B		2	29	15	11	1	3	15	15	3	4
*cva*C		2	29	15	11	1	3	15	15	3	4
*cvi*		2	29	15	11	1	3	15	15	3	4
*mcj*A	Microcin J25			4	3			4	4		
*mcj*B				4	3			4	4		
*mcj*C				4	3			4	4		
*mcj*D				4	3			4	4		
*mch*B	Microcin H47			2	1	1	3	2	2	1	1
*mchC*				2	1	1	3	2	2	1	1
*mch*I				2	1	1	3	2	2	1	1

*BAG screening results for 179 porcine E. coli representing two feeding groups associated with different ZnCl_2_ MICs are displayed*.

*ST, sequence type; HZG, high-zinc fed group; CG, control group; BAC, bacteriocin associated genes*.

Other types were only rarely detected, including colicin S4 (HZG *n* = 4; CG *n* = 1), colicin Ib (HZG, *n* = 4; CG, *n* = 2), colicin E5 (HZG, *n* = 0; CG, *n* = 2), colicin E7 (HZG, *n* = 3; CG, *n* = 2), microcin H47 (HZG, *n* = 2; CG, *n* = 1), and microcin J25 (HZG, *n* = 4; CG, *n* = 0).

Considering the pathogenic isolates investigated in this study, IMT203 (ETEC) and IMT8071 (EPEC) harbor genes encoding colicin B und M (Supplementary Table 2), while IMT19 (STEC) and the high-zinc tolerant reference strain RKI6122 possessed genes for colicin Ia.

### Occurrence of BAGs Among *E. coli* Isolates With Different Zinc Tolerance Levels

As mentioned before, the focus of our analysis was on the three different ZnCl_2_ MIC values determined for the *E. coli* collection in a previous study (22).

Of the seven 128 μg/ml ZnCl_2_ MIC isolates, two harbored BAGs. Both belonged to ST34, serotype O92:H2 and were positive for genes encoding colicin B, V, E5, and E7, while the remaining *E. coli* with 128 μg/ml ZnCl_2_ MIC did not carry any of the BAGs investigated.

One third of the isolates with MICs of 256 μg/ml ZnCl_2_ (*n* = 136, 17 STs) carried at least one bacteriocin gene (*n* = 46, 34%). In addition, colicin M, colicin S4, colicin Ib and the microcin J25 were found to be exclusively associated with isolates showing the MIC value of 256 μg/ml for ZnCl_2_ in a wide range of genomic backgrounds (Figure 1, Table 3). Isolates with a higher level of zinc tolerance (512 μg/ml MIC; *n* = 36, 7 STs) showed, by comparison, only rarely colicin encoding genes (*n* = 3, 8.33%) (Figure 1, Table 3).

## Discussion

Here we showed an analysis of *E. coli* genomes representing intestinal isolates collected from piglets fed with either a zinc-rich or common piglet diet (23) which exhibited three distinct zinc tolerance levels (22). A combination of different *in silico* analyses of the WGS data provided insights into the phylogenetic structure of these 179 porcine *E. coli* and their virulence and bacteriocin associated gene profiles. At present, there is only limited information available on the genetic diversity and relatedness of intestinal *E. coli* from pigs receiving high-zinc supplemented feed (13, 16, 23, 54).

Our results confirm that a certain phylogenetic background does not contribute to a particular zinc tolerance level in *E. coli*, since the tolerance levels obviously differed even within the STs of a single BAPS cluster (Figure 1). Despite their clearly phylogenetic heterogeneity (Figure 1), all six pathogenic porcine *E. coli* included for comparative purposes were assigned to the same “middle class” zinc tolerance level (256 μg/ml ZnCl_2_ MIC).

Serogroups frequently reported as being associated with diarrhea in pigs are O8, O138, O139, O141, O147, O149, and O157 (5, 20, 44, 55, 56). Our *E. coli* collection included a total of *n* = 23 isolates belonging to O8 (different H-types), one O149:H12 and seven O157:H43 isolates (Supplementary Table 1). Besides its association with porcine diarrhea, O157 isolates are of clinical importance for human patients, since Shiga toxin-producing *E. coli* of this particular serotype have been reported as a cause of severe foodborne illnesses (57, 58). Considering the collection investigated here, strains of this serotype were exclusively found in the CG, associated with the 256 μg/ml ZnCl_2_ MIC, harboring two to five VAGs, but all were negative for genes encoding Shiga toxins.

Isolates belonging to the O8 serogroup were detected in samples from both feeding groups, but more frequently in those representing the CG (HZG, *n* = 8; CG, *n* = 15). Interestingly, these CG isolates harbored none to two VAGs, including n=9 that are *stx*2e-positive, while Shiga toxin-encoding genes were completely absent from isolates of HZG samples.

Despite the high standards of the animal trial performed and the good health status of the animals during the whole trial period (23), our strain collection apparently included *E. coli* belonging to serotypes frequently associated with disease. In addition, isolates harboring key pathogenic markers defining pathotypes (Table 2) were identified as well, but they were less frequent among isolates representing the HZG, indicating a possible prophylactic effect of the feed additive. It also underlines the importance of the husbandry conditions to ensure a healthy gut microbiome (59), and, in line with this -to minimize the necessity of antibiotic consumption affecting the natural composition of the intestinal microbiota (60).

Furthermore, we identified serotype O26 : H11 belonging to ST21 (Supplementary Table 1), a strain background mainly reported for isolates of human and bovine origin and presumptively associated with EPEC and STEC/EHEC pathotypes (61–65). Previously, aEPEC strains of ST21-O26:H11 showed a close phylogenetic relationship to STEC/EHEC and the results of lysogenic conversion experiments using *stx*-depleted bacteriophage identified aEPEC (ST21-O26:H11) as progenitors of typical EHEC (66). Atypical EPEC (ST21-O26:H11) were also identified in our isolate collection, harboring a combination of VAGs (*eae*-positive, *stx*-negative, *bfp*-negative) meeting criteria for possible progenitor of human-pathogenic STEC/EHEC (67, 68).

While there is generally a strong association between genetic background and accessory gene content including VAGs and BAGs in our isolate collection, their occurrence and distribution were not solely lineage-specific: Some of the VAGs investigated here, for instance *iha, est*A and *est*B were present in different (Figure 1, Table 1). The EAEC associated enterotoxin EAST-1 (*ast*A) was detected in *E. coli* from both feeding groups. While it has been assumed that there is an association between this toxin and diarrhea in humans and pigs (30, 31, 69), a further study found higher *ast*A rates in *E. coli* from healthy pigs compared to isolates obtained from clinically ill pigs, questioning the particular role of *ast*A as a virulence factor in porcine *E. coli* (32). In addition, the occurrence of one or a few VAGs alone does not necessarily reflect the pathogenic potential of *E. coli*, unless the strain acquires a compatible combination of VAGs able to cause disease in a specific host species (70).

The logistic regression applied in the present study revealed a positive association between the presence of *E. coli* major diagnostic markers and the 512 μg/ml ZnCl_2_ MIC. However, whether these *E. coli* are able to cause enteral disorders in piglets needs to be further studied. The heat stable enterotoxin I encoded by *est*A, which is the most common pathogenic marker associated with the 512 μg/ml ZnCl_2_ MIC, has been reported as a cause of neonatal diarrhea in different animals. This enterotoxin seems to be associated with ETEC causing PWD as well, but rarely as the sole enterotoxin (5). However, none of the six porcine pathogenic *E. coli* variants (aEPEC, ETEC, STEC; ETEC/STEC) included in our study for comparative purposes exhibited the 512 μg/ml ZnCl_2_ MIC (Table 2), possibly suggesting that pathogenic field strains commonly lack enhanced zinc tolerance.

Following this line of thought, it seems likely that zinc does not only affect the overall composition of the porcine *E. coli* intestinal population favoring phenotypes with enhanced zinc tolerance (22, 71), but also reduces the abundance of potentially harmful (field) strains. These effects possibly provide an explanation for former observations indicating an increase in relative abundance of intestinal *Enterobacterales* among piglets fed with high amounts of zinc (72, 73), while the occurrence of PWD and detection of pathogenic bacteria seemed to decrease coincidentally (74).

Our findings might also indicate the presence of a zinc-tolerant subpopulation of *E. coli* carrying genetic marker for the intestinal pathotypes. In the environment of an animal trial with high-standard hygienic conditions, this subpopulation might not be able to proliferate well enough to cause disease, but the presence of this subpopulation indicates the importance of housing conditions, feed and water hygiene and prophylactic measures like vaccinations to maintain a healthy animal population and produce a save food product.

Since fimbriae are key features of pathogenic *E. coli* in pigs (20, 30, 75–77), we were not surprised that our collection of isolates obtained from healthy piglets lacked these particular genes (Table 1). Notably, a former study investigating 844 *E. coli* isolates from PWD-affected pig farms in Europe showed that the prevalence of genes encoding fimbriae was low even among PWD-associated ETEC (9% F4 fimbriae; 9% F18 fimbriae) from Germany (19), possibly indicating that further factors might play a role in the pathogenesis of *E. coli* in PWD.

Moreover, previous research has also shown that the bacteriophage-encoded genes *stx*_1_ and s*tx*_2_ are upregulated by DNA-damage induced activation of the SOS-response (78, 79), while zinc excess has been shown to mute the SOS-response via inhibition of *rec*A (80). In addition, zinc is also known to inhibit expression of further VAGs (81), including those promoting adherence to the gut epithelium (i.e., *esp* genes) (82). In consequence, pathogenic strains might lose their ability to harm the host, which has exemplarily been demonstrated for STEC as well as EPEC before (82).

Since bacteriocins promote the producing bacterial cells while competing for resources in a distinct ecological niche with other *E. coli* strains and closely related bacteria, they probably play an important role in structuring microbial communities residing in the gut (83). In addition, activity of particular bacteriocin-producing *E. coli* toward competing bacteria, e.g., STEC O157:H7, has been reported (84, 85). This led us to investigate if HZG-*E. coli* and/or those isolates associated with the 512 μg/ml ZnCl_2_ MIC harbor either more BAGs or a certain BAG pattern (Figure 1, Table 3). Yet, similar to the results for VAGs, BAGs seem to be less common among isolates representing the HZG and especially less common among the group of *E. coli* expressing the 512 μg/ml ZnCl_2_ MIC.

The general structural organization of the colicin encoding operons includes at least two, usually three genes (i.e., activity gene, gene encoding the immunity protein and gene encoding the lysis protein), which are commonly co-located on a large conjugative plasmid in *E. coli* (52, 86, 87). Colicins B and M are among the most common colicins in porcine *E. coli* (12, 19, 23, 88). While most of the colicins are released by cell death of the producing cell only, these colicins are among the few actively secreted bacteriocins (53) and their production might therefore be less “costly” (89). In our dataset, colicin B and M were frequently associated with isolates belonging to the CG, while rarely detected among isolates of the HZG (Table 3). For most colicins, induction is assumed to be associated with the DNA-damage induced SOS response (90). However, recent research indicated the production of biologically significant amounts of several colicins in the absence of such stress (91). Furthermore, colicin M was found to be secreted despite the absence of the SOS box believed to regulate its production (88). Consequently, higher detection rates of genes encoding colicin M might be a result from its less severe “production consequences” for the individual cell, since cell death is not a prerequisite for its release. Interestingly, genes encoding colicin BM were found in BAPS cluster I, II, IV and V (Figure 1), but all these isolates lack zinc tolerance above the mid-range 256 μg/ml ZnCl_2_ MIC (Supplementary Table 1). The complete absence of colicin BM genes in isolates associated with enhanced zinc tolerance (isolates distributed within BAPS cluster I, II, III, VI and VII) suggests that co-occurrence of enhanced zinc tolerance with colicin BM production lacks a beneficial outcome for intestinal *E. coli* in piglets fed with high amounts of zinc. However, more investigation on this subject is clearly needed.

Contrarily, the operon encoding microcin V was more associated with isolates from the HZG than the CG, however it was also rarely associated with isolates with a 512 μg/ml ZnCl_2_ MIC. The expression of the immunity protein (Cvi) of this operon depends on the presence of iron [reviewed in (53)], since the *cvi* promoter region is associated with a previously identified binding site for the ferric uptake regulation protein (Fur) (53, 92). Interestingly, zinc excess was reported to increase the bacterial demand for iron (93), and microcin V activation might increase the chances of bacteria to benefit from iron released by dead competing populations. However, more research on functional interactions of bacteria under zinc-induced stress is needed, which left speculation only considering the interlinkages of microcin V expression and zinc excess.

## Conclusion

Our analysis comprising 179 *E. coli* obtained from piglets of two different feeding groups using Bayesian analysis of the genomes showed a population structure (BAPS) which lacked an association between a particular zinc tolerance level and a phylogenetic cluster or even branch for both, isolates belonging to the HZG and CG, suggesting that zinc tolerance is not a characteristic of a particular phylogenetic background. In addition, detection rates for genes and operons associated with virulence (VAG) and bacteriocins (BAG) were lower in isolates originating from the HZG (41 vs. 65% and 22 vs. 35%, *p* < 0.001 and *p* = 0.002, resp.), indicating an effect of high-zinc supplementation of the piglets' diet on the occurrences of these genes among intestinal *E. coli* populations.

This effect seems to be more even more important considering *E. coli* harboring genes defining distinct pathotypes associated with intestinal disease, i.e., enterotoxigenic, enteropathogenic and Shiga toxin-producing *E. coli* (ETEC, EPEC and STEC), which constituted only 1% of the isolates belonging to the HZG but 14% of those from the CG, supporting previous observations that high zinc supplemented diets for piglets were associated with a decrease of PWD.

## Data Availability Statement

The datasets presented in this study can be found in online repositories. The names of the repository/repositories and accession number(s) can be found in the article/Supplementary Material. Illumina raw read data for 186 *E. coli* are available at NCBI Bioproject ID PRJNA552271.

## Ethics Statement

The representative set of *E. coli* isolates investigated here was selected based on a previous feeding trial (23) carried out in accordance with the principles of the Basel Declaration following the institutional and national guidelines for the care and use of animals. The animal study was reviewed and approved by the local state office of occupational health and technical safety Landesamt für Gesundheit und Soziales, Berlin (LaGeSo Reg. Nr. 0296/13) as described before (23).

## Author Contributions

LW, AL-B, and AB designed the project. VJ and BW conceived and designed the experiments. IE sequenced the isolates. VJ performed laboratory analysis. VJ, BW, LE, AB, TS, FG, and RM analyzed the data. VJ, AB, BW, and LW wrote the article. All authors have read and approved the final draft of the manuscript. All authors contributed to the article and approved the submitted version.

## Conflict of Interest

The authors declare that the research was conducted in the absence of any commercial or financial relationships that could be construed as a potential conflict of interest.
