# Supplementary material for: High-Zinc Supplementation of Weaned Piglets Affects Frequencies of Virulence and Bacteriocin Associated Genes Among Intestinal Escherichia coli Populations
Source: Front Vet Sci. 2020 Dec 16;7:614513. doi: 10.3389/fvets.2020.614513 (PMC7772137; doi:10.3389/fvets.2020.614513)
Supplement: Supplementary file 4 [file Data_Sheet_1.PDF]

Supplemental figure 1|
Summary of amino acid (aa) identity of putative bacteriocins and cognate immunity and lysin genes identified *in silico.*

cba – Colicin B activity gene [UniProtKB: P05819]

n= 19 isolates

Amino acid (aa) identity of <u>Colicin B</u> [%]

Isolate-ID	P05819 (REF)	3873	4865	3888	3887	3886	3885	3049	3048	3046	3041	3038	4137	3923	3307	3304	3302	3266	4707
3873	99.8																		
4865	99.8	99.6																	
3888	99.8	99.6	100.0																
3887	99.8	99.6	100.0	100.0															
3886	99.8	99.6	100.0	100.0	100.0														
3885	99.8	99.6	100.0	100.0	100.0	100.0													
3049	99.8	99.6	100.0	100.0	100.0	100.0	100.0												
3048	99.8	99.6	100.0	100.0	100.0	100.0	100.0	100.0											
3046	99.8	99.6	100.0	100.0	100.0	100.0	100.0	100.0	100.0										
3041	99.8	99.6	100.0	100.0	100.0	100.0	100.0	100.0	100.0	100.0									
3038	99.8	99.6	100.0	100.0	100.0	100.0	100.0	100.0	100.0	100.0	100.0								
4137	99.6	99.4	99.4	99.4	99.4	99.4	99.4	99.4	99.4	99.4	99.4	99.4							
3923	99.8	99.6	99.6	99.6	99.6	99.6	99.6	99.6	99.6	99.6	99.6	99.6	99.4						
3307	99.6	99.4	99.4	99.4	99.4	99.4	99.4	99.4	99.4	99.4	99.4	99.4	100.0	99.4					
3304	99.6	99.4	99.4	99.4	99.4	99.4	99.4	99.4	99.4	99.4	99.4	99.4	100.0	99.4	100.0				
3302	99.6	99.4	99.4	99.4	99.4	99.4	99.4	99.4	99.4	99.4	99.4	99.4	100.0	99.4	100.0	100.0			
3266	99.8	99.6	99.6	99.6	99.6	99.6	99.6	99.6	99.6	99.6	99.6	99.6	99.4	100.0	99.4	99.4	99.4		
4707	90.0	90.0	89.8	89.8	89.8	89.8	89.8	89.8	89.8	89.8	89.8	89.8	89.6	90.0	89.6	89.6	89.6	90.0	
4696	90.0	90.0	89.8	89.8	89.8	89.8	89.8	89.8	89.8	89.8	89.8	89.8	89.6	90.0	89.6	89.6	89.6	90.0	100.0

cbi – Colicin B immunity gene [UniProtKB: P22426]

n= 28 isolates

0 | 98.0 | 99.99 | 100.0

aa sequence idendity (%)

Isolate-ID	P22426 (REF)	4107	3333	6215	3363	3873	4865	3888	3887	3886	3885	3049	3048	3046	3041	3038	4137	3923	3307	3304	3302	3266	4707	4696	4138	4932	6210	6212
4107	98.3																											
3333	98.3	100.0																										
6215	98.3	100.0	100.0																									
3363	98.3	100.0	100.0	100.0																								
3873	100.0	100.0	100.0	100.0	100.0																							
4865	98.9	98.3	98.3	98.3	98.3	98.9																						
3888	98.9	98.3	98.3	98.3	98.3	98.9	100.0																					
3887	98.9	98.3	98.3	98.3	98.3	98.9	100.0	100.0																				
3886	98.9	98.3	98.3	98.3	98.3	98.9	100.0	100.0	100.0																			
3885	98.9	98.3	98.3	98.3	98.3	98.9	100.0	100.0	100.0	100.0																		
3049	98.9	98.3	98.3	98.3	98.3	98.9	100.0	100.0	100.0	100.0	100.0																	
3048	98.9	98.3	98.3	98.3	98.3	98.9	100.0	100.0	100.0	100.0	100.0	100.0																
3046	98.9	98.3	98.3	98.3	98.3	98.9	100.0	100.0	100.0	100.0	100.0	100.0	100.0															
3041	98.9	98.3	98.3	98.3	98.3	98.9	100.0	100.0	100.0	100.0	100.0	100.0	100.0	100.0														
3038	98.9	98.3	98.3	98.3	98.3	98.9	100.0	100.0	100.0	100.0	100.0	100.0	100.0	100.0	100.0													
4137	98.9	98.3	98.3	98.3	98.3	98.9	98.9	98.9	98.9	98.9	98.9	98.9	98.9	98.9	98.9	98.9												
3923	100.0	98.3	98.3	98.3	98.3	100.0	98.9	98.9	98.9	98.9	98.9	98.9	98.9	98.9	98.9	98.9	98.9											
3307	98.9	98.3	98.3	98.3	98.3	98.9	98.9	98.9	98.9	98.9	98.9	98.9	98.9	98.9	98.9	98.9	98.9	98.9										
3304	98.9	98.3	98.3	98.3	98.3	98.9	98.9	98.9	98.9	98.9	98.9	98.9	98.9	98.9	98.9	98.9	98.9	98.9	100.0									
3302	98.9	98.3	98.3	98.3	98.3	98.9	98.9	98.9	98.9	98.9	98.9	98.9	98.9	98.9	98.9	98.9	98.9	98.9	100.0	100.0								
3266	100.0	98.3	98.3	98.3	98.3	100.0	98.9	98.9	98.9	98.9	98.9	98.9	98.9	98.9	98.9	98.9	98.9	98.9	98.9	98.9	98.9							
4707	98.9	98.3	98.3	98.3	98.3	98.9	98.9	98.9	98.9	98.9	98.9	98.9	98.9	98.9	98.9	98.9	98.9	98.9	98.9	98.9	98.9	98.9						
4696	98.9	98.3	98.3	98.3	98.3	98.9	98.9	98.9	98.9	98.9	98.9	98.9	98.9	98.9	98.9	98.9	98.9	98.9	98.9	98.9	98.9	98.9	98.9					
4138	100.0	98.3	98.3	98.3	98.3	100.0	98.9	98.9	98.9	98.9	98.9	98.9	98.9	98.9	98.9	98.9	98.9	98.9	98.9	98.9	98.9	100.0	98.9	98.9				
4932	100.0	98.3	98.3	98.3	98.3	100.0	98.9	98.9	98.9	98.9	98.9	98.9	98.9	98.9	98.9	98.9	98.9	98.9	98.9	98.9	98.9	100.0	98.9	98.9	100.0			
6210	100.0	98.3	98.3	98.3	98.3	100.0	98.9	98.9	98.9	98.9	98.9	98.9	98.9	98.9	98.9	98.9	98.9	98.9	98.9	98.9	98.9	100.0	98.9	98.9	100.0	100.0		
6212	100.0	98.3	98.3	98.3	98.3	100.0	98.9	98.9	98.9	98.9	98.9	98.9	98.9	98.9	98.9	98.9	98.9	98.9	98.9	98.9	98.9	98.3	98.9	98.9	100.0	100.0	100.0	
4082	100.0	98.3	98.3	98.3	98.3	100.0	98.9	98.9	98.9	98.9	98.9	98.9	98.9	98.9	98.9	98.9	98.9	98.9	98.9	98.9	98.9	100.0	98.9	98.9	100.0	100.0	100.0	100.0

cma – Colicin M activity gene [UniProtKB: P05820]

Amino acid (aa) identity of Colicin M [%]

n= 26 isolates

Isolate-ID	P05820 (REF)	6212	6210	4932	4865	4138	4082	3888	3887	3886	3885	3049	3048	3046	3041	3038	3923	3266	6215	4107	3873	3363	3333	4137	3307	3304
6212	100.0																									
6210	100.0	100.0																								
4932	100.0	100.0	100.0																							
4865	100.0	100.0	100.0	100.0																						
4138	100.0	100.0	100.0	100.0	100.0																					
4082	100.0	100.0	100.0	100.0	100.0	100.0																				
3888	100.0	100.0	100.0	100.0	100.0	100.0	100.0																			
3887	100.0	100.0	100.0	100.0	100.0	100.0	100.0	100.0																		
3886	100.0	100.0	100.0	100.0	100.0	100.0	100.0	100.0	100.0																	
3885	100.0	100.0	100.0	100.0	100.0	100.0	100.0	100.0	100.0	100.0																
3049	100.0	100.0	100.0	100.0	100.0	100.0	100.0	100.0	100.0	100.0	100.0															
3048	100.0	100.0	100.0	100.0	100.0	100.0	100.0	100.0	100.0	100.0	100.0	100.0														
3046	100.0	100.0	100.0	100.0	100.0	100.0	100.0	100.0	100.0	100.0	100.0	100.0	100.0													
3041	100.0	100.0	100.0	100.0	100.0	100.0	100.0	100.0	100.0	100.0	100.0	100.0	100.0	100.0												
3038	100.0	100.0	100.0	100.0	100.0	100.0	100.0	100.0	100.0	100.0	100.0	100.0	100.0	100.0	100.0											
3923	99.6	99.6	99.6	99.6	99.6	99.6	99.6	99.6	99.6	99.6	99.6	99.6	99.6	99.6	99.6	99.6										
3266	99.6	99.6	99.6	99.6	99.6	99.6	99.6	99.6	99.6	99.6	99.6	99.6	99.6	99.6	99.6	99.6	100.0									
6215	99.3	99.3	99.3	99.3	99.3	99.3	99.3	99.3	99.3	99.3	99.3	99.3	99.3	99.3	99.3	99.3	98.9	98.9								
4107	99.3	99.3	99.3	99.3	99.3	99.3	99.3	99.3	99.3	99.3	99.3	99.3	99.3	99.3	99.3	99.3	98.9	98.9	100.0							
3873	99.3	99.3	99.3	99.3	99.3	99.3	99.3	99.3	99.3	99.3	99.3	99.3	99.3	99.3	99.3	99.3	98.9	98.9	100.0	100.0						
3363	99.3	99.3	99.3	99.3	99.3	99.3	99.3	99.3	99.3	99.3	99.3	99.3	99.3	99.3	99.3	99.3	98.9	98.9	100.0	100.0	100.0					
3333	99.3	99.3	99.3	99.3	99.3	99.3	99.3	99.3	99.3	99.3	99.3	99.3	99.3	99.3	99.3	99.3	98.9	98.9	100.0	100.0	100.0	100.0				
4137	98.9	98.9	98.9	98.9	98.9	98.9	98.9	98.9	98.9	98.9	98.9	98.9	98.9	98.9	98.9	98.9	98.5	98.5	98.2	98.2	98.2	98.2	98.2			
3307	98.9	98.9	98.9	98.9	98.9	98.9	98.9	98.9	98.9	98.9	98.9	98.9	98.9	98.9	98.9	98.9	98.5	98.5	98.2	98.2	98.2	98.2	98.2	100.0		
3304	98.9	98.9	98.9	98.9	98.9	98.9	98.9	98.9	98.9	98.9	98.9	98.9	98.9	98.9	98.9	98.9	98.5	98.5	98.2	98.2	98.2	98.2	98.2	100.0	100.0	
3302	98.9	98.9	98.9	98.9	98.9	98.9	98.9	98.9	98.9	98.9	98.9	98.9	98.9	98.9	98.9	98.9	98.5	98.5	98.2	98.2	98.2	98.2	98.2	100.0	100.0	100.0

REF — Isolate-ID

Legend: 0 — 98.0 — 99.99 — 100.0

aa sequence idendity (%)

cmi – Colicin M immunity gene [UniProtKB: P18002]

Isolate-ID	P18002 (REF)	6212	4138	3266	3873	3923	4082	6210	4932	4107	3333	6215	3363	3873	4137	3304	3302	3307	3041	3048	3049	3888	3887	3886	3885	3038
6212	99.3																									
4138	99.3	100.0																								
3266	99.3	100.0	100.0																							
3873	99.3	100.0	100.0	100.0																						
3923	99.3	100.0	100.0	100.0	100.0																					
4082	99.3	100.0	100.0	100.0	100.0	100.0																				
6210	99.3	100.0	100.0	100.0	100.0	100.0	100.0																			
4932	99.3	100.0	100.0	100.0	100.0	100.0	100.0	100.0																		
4107	99.3	100.0	100.0	100.0	100.0	100.0	100.0	100.0	100.0																	
3333	99.3	100.0	100.0	100.0	100.0	100.0	100.0	100.0	100.0	100.0																
6215	99.3	100.0	100.0	100.0	100.0	100.0	100.0	100.0	100.0	100.0	100.0															
3363	99.3	100.0	100.0	100.0	100.0	100.0	100.0	100.0	100.0	100.0	100.0	100.0														
4137	99.3	100.0	100.0	100.0	100.0	100.0	100.0	100.0	100.0	100.0	100.0	100.0	100.0													
3304	99.3	100.0	100.0	100.0	100.0	100.0	100.0	100.0	100.0	100.0	100.0	100.0	100.0	100.0												
3302	99.3	100.0	100.0	100.0	100.0	100.0	100.0	100.0	100.0	100.0	100.0	100.0	100.0	100.0	100.0											
3307	99.3	100.0	100.0	100.0	100.0	100.0	100.0	100.0	100.0	100.0	100.0	100.0	100.0	100.0	100.0	100.0										
3041	99.3	100.0	100.0	100.0	100.0	100.0	100.0	100.0	100.0	100.0	100.0	100.0	100.0	100.0	100.0	100.0	100.0									
3048	99.2	100.0	100.0	100.0	100.0	100.0	100.0	100.0	100.0	100.0	100.0	100.0	100.0	100.0	100.0	100.0	100.0	100.0								
3049	100.0	100.0	100.0	100.0	100.0	100.0	100.0	100.0	100.0	100.0	100.0	100.0	100.0	100.0	100.0	100.0	100.0	100.0	100.0							
3888	99.3	100.0	100.0	100.0	100.0	100.0	100.0	100.0	100.0	100.0	100.0	100.0	100.0	100.0	100.0	100.0	100.0	100.0	100.0	100.0						
3887	99.3	100.0	100.0	100.0	100.0	100.0	100.0	100.0	100.0	100.0	100.0	100.0	100.0	100.0	100.0	100.0	100.0	100.0	100.0	100.0	100.0					
3886	99.3	100.0	100.0	100.0	100.0	100.0	100.0	100.0	100.0	100.0	100.0	100.0	100.0	100.0	100.0	100.0	100.0	100.0	100.0	100.0	100.0	100.0				
3885	99.3	100.0	100.0	100.0	100.0	100.0	100.0	100.0	100.0	100.0	100.0	100.0	100.0	100.0	100.0	100.0	100.0	100.0	100.0	100.0	100.0	100.0	100.0			
3038	99.3	100.0	100.0	100.0	100.0	100.0	100.0	100.0	100.0	100.0	100.0	100.0	100.0	100.0	100.0	100.0	100.0	100.0	100.0	100.0	100.0	100.0	100.0	100.0		
4865	99.3	100.0	100.0	100.0	100.0	100.0	100.0	100.0	100.0	100.0	100.0	100.0	100.0	100.0	100.0	100.0	100.0	100.0	100.0	100.0	100.0	100.0	100.0	100.0	100.0	
3046	99.3	100.0	100.0	100.0	100.0	100.0	100.0	100.0	100.0	100.0	100.0	100.0	100.0	100.0	100.0	100.0	100.0	100.0	100.0	100.0	100.0	100.0	100.0	100.0	100.0	100.0

REF — Isolate-ID

Amino acid (aa) identity of <u>Colicin S4</u> [%]

n= 5 isolates

csa – Colicin S4 activity gene [UniProtKB: Q9XB47]

Isolate-ID	Q9XB47 (REF)	3105	3183	4758	4766
3105	98.8				
3183	98.8	100.0			
4758	97.8	97.4	97.4		
4766	97.8	97.4	97.4	100.0	
4769	97.8	97.4	97.4	100.0	100.0

csi – Colicin S4 immunity gene [UniProtKB: Q9XB46]

Isolate-ID	Q9XB46 (REF)	3105	3183	4758	4766
3105	99.4				
3183	99.4	100.0			
4758	96.6	97.2	97.2		
4766	96.6	97.2	97.2	100.0	
4769	96.6	97.2	97.2	100.0	100.0

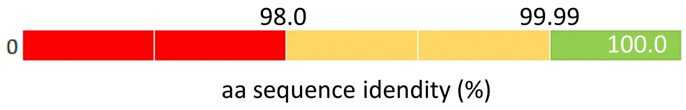

csl – Colicin S4 lysin gene [UniProtKB: Q9XB45]

Isolate-ID	Q9XB45 (REF)	3105	3183	4758	4766
3105	100.0				
3183	100.0	100.0			
4758	100.0	100.0	100.0		
4766	100.0	100.0	100.0	100.0	
4769	100.0	100.0	100.0	100.0	100.0

cia – Colicin Ia activity gene [UniProtKB: P06716]

Amino acid (aa) identity of Colicin Ia [%]

n= 22 isolates

Isolate-ID	P06716 (REF)	6213	4769	4766	4758	4729	3283	6212	6210	4932	4138	4082	4865	3888	3887	3886	3885	3049	3048	3046	3041	3038
6213	99.7																					
4769	99.7	100																				
4766	99.7	100	100																			
4758	99.7	100	100	100																		
4729	99.7	100	100	100	100																	
3283	99.7	100	100	100	100	100																
6212	99.5	99.8	99.8	99.8	99.8	99.8	99.8															
6210	99.5	99.8	99.8	99.8	99.8	99.8	99.8	100														
4932	99.5	99.8	99.8	99.8	99.8	99.8	99.8	100	100													
4138	99.5	99.8	99.8	99.8	99.8	99.8	99.8	100	100	100												
4082	99.5	99.8	99.8	99.8	99.8	99.8	99.8	100	100	100	100											
4865	99.5	99.8	99.8	99.8	99.8	99.8	99.8	99.7	99.7	99.7	99.7	99.7										
3888	99.5	99.8	99.8	99.8	99.8	99.8	99.8	99.7	99.7	99.7	99.7	99.7	100									
3887	99.5	99.8	99.8	99.8	99.8	99.8	99.8	99.7	99.7	99.7	99.7	99.7	100	100								
3886	99.5	99.8	99.8	99.8	99.8	99.8	99.8	99.7	99.7	99.7	99.7	99.7	100	100	100							
3885	99.5	99.8	99.8	99.8	99.8	99.8	99.8	99.7	99.7	99.7	99.7	99.7	100	100	100	100						
3049	99.5	99.8	99.8	99.8	99.8	99.8	99.8	99.7	99.7	99.7	99.7	99.7	100	100	100	100	100					
3048	99.5	99.8	99.8	99.8	99.8	99.8	99.8	99.7	99.7	99.7	99.7	99.7	100	100	100	100	100	100				
3046	99.5	99.8	99.8	99.8	99.8	99.8	99.8	99.7	99.7	99.7	99.7	99.7	100	100	100	100	100	100	100			
3041	99.5	99.8	99.8	99.8	99.8	99.8	99.8	99.7	99.7	99.7	99.7	99.7	100	100	100	100	100	100	100	100		
3038	99.5	99.8	99.8	99.8	99.8	99.8	99.8	99.7	99.7	99.7	99.7	99.7	100	100	100	100	100	100	100	100	100	
3802	99.7	100	100	100	100	100	100	99.8	99.8	99.8	99.8	99.8	99.8	99.8	99.8	99.8	99.8	99.8	99.8	99.8	99.8	99.8

REF — Isolate-ID

Legend: 0 — 98.0 — 99.99 — 100.0

aa sequence idendity (%)

iia – Colicin Ia immunity gene [UniProtKB: Q46741]

Isolate-ID	Q46741 (REF)	4138	6212	4082	4932	6210	4758	3038	6213	3283	4729	4865	3802	4769	4766	3049	3048	3885	3046	3886	3887	3888
4138	100.0																					
6212	100.0	100.0																				
4082	100.0	100.0	100.0																			
4932	100.0	100.0	100.0	100.0																		
6210	99.1	99.1	99.1	99.1	99.1																	
4758	99.1	99.1	99.1	99.1	99.1	98.2																
3038	99.1	99.1	99.1	99.1	99.1	98.2	100.0															
6213	99.1	99.1	99.1	99.1	99.1	98.2	100.0	100.0														
3283	99.1	99.1	99.1	99.1	99.1	98.2	100.0	100.0	100.0													
4729	99.1	99.1	99.1	99.1	99.1	98.2	100.0	100.0	100.0	100.0												
4865	99.1	99.1	99.1	99.1	99.1	98.2	100.0	100.0	100.0	100.0	100.0											
3802	99.1	99.1	99.1	99.1	99.1	98.2	100.0	100.0	100.0	100.0	100.0	100.0										
4769	99.1	99.1	99.1	99.1	99.1	98.2	100.0	100.0	100.0	100.0	100.0	100.0	100.0									
4766	99.1	99.1	99.1	99.1	99.1	98.2	100.0	100.0	100.0	100.0	100.0	100.0	100.0	100.0								
3049	99.1	99.1	99.1	99.1	99.1	98.2	100.0	100.0	100.0	100.0	100.0	100.0	100.0	100.0	100.0							
3048	99.1	99.1	99.1	99.1	99.1	98.2	100.0	100.0	100.0	100.0	100.0	100.0	100.0	100.0	100.0	100.0						
3885	99.1	99.1	99.1	99.1	99.1	98.2	100.0	100.0	100.0	100.0	100.0	100.0	100.0	100.0	100.0	100.0	100.0					
3046	99.1	99.1	99.1	99.1	99.1	98.2	100.0	100.0	100.0	100.0	100.0	100.0	100.0	100.0	100.0	100.0	100.0	100.0				
3886	99.1	99.1	99.1	99.1	99.1	98.2	100.0	100.0	100.0	100.0	100.0	100.0	100.0	100.0	100.0	100.0	100.0	100.0	100.0			
3887	99.1	99.1	99.1	99.1	99.1	98.2	100.0	100.0	100.0	100.0	100.0	100.0	100.0	100.0	100.0	100.0	100.0	100.0	100.0	100.0		
3888	99.1	99.1	99.1	99.1	99.1	98.2	100.0	100.0	100.0	100.0	100.0	100.0	100.0	100.0	100.0	100.0	100.0	100.0	100.0	100.0	100.0	
3041	99.1	99.1	99.1	99.1	99.1	98.2	100.0	100.0	100.0	100.0	100.0	100.0	100.0	100.0	100.0	100.0	100.0	100.0	100.0	100.0	100.0	100.0

REF — Isolate-ID

Amino acid (aa) identity of <u>Colicin Ib</u> [%]

n= 6 isolates

***cib* –** Colicin Ib activity gene [UniProtKB: P04479]

Isolate-ID	P04479 (REF)	3283	3855	4887	4889	4898
3283	95.7					
3855	98.3	97.4				
4887	98.3	97.4	100			
4889	98.3	97.4	100	100		
4898	98.3	97.4	100	100	100	
6213	95.7	100	97.4	97.4	97.4	97.4

0 | 98.0 | 99.99 | 100.0

aa sequence idendity (%)

***iib* –** Colicin Ib immunity gene [UniProtKB: H9XP55]

Isolate-ID	H9XP55 (REF)	3283	3855	4887	4889	4898
3283	95.7					
3855	98.3	97.4				
4887	98.3	97.4	100			
4889	98.3	97.4	100	100		
4898	98.3	97.4	100	100	100	
6213	95.7	100	97.4	97.4	97.4	97.4

Amino acid (aa) identity of Microcin V [%]

n= 18 isolates

*cva*A – Microcin V activity gene [UniProtKB: P22519]

Isolate-ID	P22519 (REF)	4881	3800	3799	3080	6215	4107	3363	3333	4898	4889	4887	4816	3855	4707	4696		3278
4881	100																	
3800	100	100																
3799	100	100	100															
3080	100	100	100	100														
6215	99.6	99.6	99.6	99.6	99.6													
4107	99.6	99.6	99.6	99.6	99.6	100												
3363	99.6	99.6	99.6	99.6	99.6	100	100											
3333	99.6	99.6	99.6	99.6	99.6	100	100	100										
4898	99.3	99.3	99.3	99.3	99.3	99.4	99.4	99.4	99.4									
4889	99.3	99.3	99.3	99.3	99.3	99.4	99.4	99.4	99.4	100								
4887	99.3	99.3	99.3	99.3	99.3	99.4	99.4	99.4	99.4	100	100							
4816	99.3	99.3	99.3	99.3	99.3	99.4	99.4	99.4	99.4	100	100	100						
3855	99.3	99.3	99.3	99.3	99.3	99.4	99.4	99.4	99.4	100	100	100	100					
4707	95.4	95.4	95.4	95.4	95.4	95.6	95.6	95.6	95.6	95.6	95.6	95.6	95.6	95.6				
4696	95.4	95.4	95.4	95.4	95.4	95.6	95.6	95.6	95.6	95.6	95.6	95.6	95.6	95.6	100			
3798	89.8	89.8	89.8	89.8	89.8	89.7	89.7	89.7	89.7	89.7	89.7	89.7	89.7	89.7	92.1	92.1		
3278	89.8	89.8	89.8	89.8	89.8	89.7	89.7	89.7	89.7	89.7	89.7	89.7	89.7	89.7	92.1	92.1	100	
3082	89.8	89.8	89.8	89.8	89.8	89.7	89.7	89.7	89.7	89.7	89.7	89.7	89.7	89.7	92.1	92.1	100	100

Legend: 0 – 98.0 – 99.99 – 100.0

aa sequence idendity (%)

*cva*B – Microcin V activity gene [UniProtKB: P22520]

Isolate-ID	P22520 (REF)	4881	3800	3799	3080	6215	4107	3363	3333	4898	4889	4887	4816	3855	4707	4696	3798	
4881	100.0																	
3800	100.0	100.0																
3799	100.0	100.0	100.0															
3080	100.0	100.0	100.0	100.0														
6215	99.6	99.6	99.6	99.6	99.6													
4107	99.6	99.6	99.6	99.6	99.6	100.0												
3363	99.6	99.6	99.6	99.6	99.6	100.0	100.0											
3333	99.6	99.6	99.6	99.6	99.6	100.0	100.0	100.0										
4898	99.3	99.3	99.3	99.3	99.3	99.4	99.4	99.4	99.4									
4889	99.3	99.3	99.3	99.3	99.3	99.4	99.4	99.4	99.4	100.0								
4887	99.3	99.3	99.3	99.3	99.3	99.4	99.4	99.4	99.4	100.0	100.0							
4816	99.3	99.3	99.3	99.3	99.3	99.4	99.4	99.4	99.4	100.0	100.0	100.0						
3855	99.3	99.3	99.3	99.3	99.3	99.4	99.4	99.4	99.4	100.0	100.0	100.0	100.0					
4707	95.4	95.4	95.4	95.4	95.4	95.6	95.6	95.6	95.6	95.6	95.6	95.6	95.6	95.6				
4696	95.4	95.4	95.4	95.4	95.4	95.6	95.6	95.6	95.6	95.6	95.6	95.6	95.6	95.6	100.0			
3798	89.8	89.8	89.8	89.8	89.8	89.7	89.7	89.7	89.7	89.7	89.7	89.7	89.7	89.7	92.1	92.1		
3278	89.8	89.8	89.8	89.8	89.8	89.7	89.7	89.7	89.7	89.7	89.7	89.7	89.7	89.7	92.1	92.1	100.0	
3082	89.8	89.8	89.8	89.8	89.8	89.7	89.7	89.7	89.7	89.7	89.7	89.7	89.7	89.7	92.1	92.1	100.0	100.0

cvaC – Microcin V activity gene [UniProtKB: P22522]

Amino acid (aa) identity of <u>Microcin V</u> [%]

n= 18 isolates

Isolate-ID	P22522 (REF)	4881	3800	3799	3080	6215	4107	3363	3333	4898	4889	4887	4816	3855	4707	4696	3798	3278
4881	100.0																	
3800	100.0	100.0																
3799	100.0	100.0	100.0															
3080	100.0	100.0	100.0	100.0														
6215	99.6	99.6	99.6	99.6	99.6													
4107	99.6	99.6	99.6	99.6	99.6	100.0												
3363	99.6	99.6	99.6	99.6	99.6	100.0	100.0											
3333	99.6	99.6	99.6	99.6	99.6	100.0	100.0	100.0										
4898	99.3	99.3	99.3	99.3	99.3	99.4	99.4	99.4	99.4									
4889	99.3	99.3	99.3	99.3	99.3	99.4	99.4	99.4	99.4	100.0								
4887	99.3	99.3	99.3	99.3	99.3	99.4	99.4	99.4	99.4	100.0	100.0							
4816	99.3	99.3	99.3	99.3	99.3	99.4	99.4	99.4	99.4	100.0	100.0	100.0						
3855	99.3	99.3	99.3	99.3	99.3	99.4	99.4	99.4	99.4	100.0	100.0	100.0	100.0					
4707	95.4	95.4	95.4	95.4	95.4	95.6	95.6	95.6	95.6	95.6	95.6	95.6	95.6	95.6				
4696	95.4	95.4	95.4	95.4	95.4	95.6	95.6	95.6	95.6	95.6	95.6	95.6	95.6	95.6	100.0			
3798	89.8	89.8	89.8	89.8	89.8	89.7	89.7	89.7	89.7	89.7	89.7	89.7	89.7	89.7	92.1	92.1		
3278	89.8	89.8	89.8	89.8	89.8	89.7	89.7	89.7	89.7	89.7	89.7	89.7	89.7	89.7	92.1	92.1	100.0	
3082	89.8	89.8	89.8	89.8	89.8	89.7	89.7	89.7	89.7	89.7	89.7	89.7	89.7	89.7	92.1	92.1	100.0	100.0

0 | 98.0 | 99.99 | 100.0

aa sequence idendity (%)

cvi – Microcin V immunity gene [UniProtKB: P22521]

Isolate-ID	P22521 (REF)	4881	3800	3799	3080	6215	4107	3363	3333	4898	4889	4887	4816	3855	4707	4696	3798	3278
4881	100.0																	
3800	100.0	100.0																
3799	100.0	100.0	100.0															
3080	100.0	100.0	100.0	100.0														
6215	100.0	100.0	100.0	100.0	100.0													
4107	100.0	100.0	100.0	100.0	100.0	100.0												
3363	100.0	100.0	100.0	100.0	100.0	100.0	100.0											
3333	100.0	100.0	100.0	100.0	100.0	100.0	100.0	100.0										
4898	100.0	100.0	100.0	100.0	100.0	100.0	100.0	100.0	100.0									
4889	100.0	100.0	100.0	100.0	100.0	100.0	100.0	100.0	100.0	100.0								
4887	100.0	100.0	100.0	100.0	100.0	100.0	100.0	100.0	100.0	100.0	100.0							
4816	100.0	100.0	100.0	100.0	100.0	100.0	100.0	100.0	100.0	100.0	100.0	100.0						
3855	100.0	100.0	100.0	100.0	100.0	100.0	100.0	100.0	100.0	100.0	100.0	100.0	100.0					
4707	100.0	100.0	100.0	100.0	100.0	100.0	100.0	100.0	100.0	100.0	100.0	100.0	100.0	100.0				
4696	100.0	100.0	100.0	100.0	100.0	100.0	100.0	100.0	100.0	100.0	100.0	100.0	100.0	100.0	100.0			
3798	100.0	100.0	100.0	100.0	100.0	100.0	100.0	100.0	100.0	100.0	100.0	100.0	100.0	100.0	100.0	100.0		
3278	100.0	100.0	100.0	100.0	100.0	100.0	100.0	100.0	100.0	100.0	100.0	100.0	100.0	100.0	100.0	100.0	100.0	
3082	100.0	100.0	100.0	100.0	100.0	100.0	100.0	100.0	100.0	100.0	100.0	100.0	100.0	100.0	100.0	100.0	100.0	100.0

Amino acid (aa) identity of Colicin E1 [%]

n= 13 isolates

cea – Colicin E1 activity gene [UniProtKB: P02978]

Isolate-ID	P02978 (REF)	3798	3278	3082	3873	4898	4889	4887	4816	4769	4766	4758	3855
3798	99.8												
3278	99.8	100.0											
3082	99.8	100.0	100.0										
3873	99.6	99.8	99.8	99.8									
4898	85.5	85.7	85.7	85.7	85.5								
4889	85.5	85.7	85.7	85.7	85.5	100.0							
4887	85.5	85.7	85.7	85.7	85.5	100.0	100.0						
4816	85.5	85.7	85.7	85.7	85.5	100.0	100.0	100.0					
4769	85.5	85.7	85.7	85.7	85.5	100.0	100.0	100.0	100.0				
4766	85.5	85.7	85.7	85.7	85.5	100.0	100.0	100.0	100.0	100.0			
4758	85.5	85.7	85.7	85.7	85.5	100.0	100.0	100.0	100.0	100.0	100.0		
3855	85.5	85.7	85.7	85.7	85.5	100.0	100.0	100.0	100.0	100.0	100.0	100.0	
3181	85.9	85.9	85.9	85.9	85.5	100.0	100.0	100.0	100.0	100.0	100.0	100.0	100.0

imm – Colicin E1 immunity gene [UniProtKB: P02985]

Isolate-ID	P02985 (REF)	3798	3082	3873	3076	4766	4758	4769	4889	3855	4816	4898	4887
3798	100.0												
3082	100.0	100.0											
3873	99.1	99.1	99.1										
3076	98.9	98.9	98.9	100.0									
4766	92.9	92.9	92.9	93.8	93.6								
4758	92.9	92.9	92.9	93.8	93.6	100.0							
4769	92.9	92.9	92.9	93.8	93.6	100.0	100.0						
4889	92.9	92.9	92.9	93.8	93.6	100.0	100.0	100.0					
3855	92.9	92.9	92.9	93.8	93.6	100.0	100.0	100.0	100.0				
4816	92.9	92.9	92.9	93.8	93.6	100.0	100.0	100.0	100.0	100.0			
4898	92.9	92.9	92.9	93.8	93.6	100.0	100.0	100.0	100.0	100.0	100.0		
4887	92.9	92.9	92.9	93.8	93.6	100.0	100.0	100.0	100.0	100.0	100.0	100.0	
3181	92.9	92.9	92.9	93.8	93.6	100.0	100.0	100.0	100.0	100.0	100.0	100.0	100.0

lys – Colicin E1 lysin gene [UniProtKB: P05821]

Isolate-ID	P05821 (REF)	3798	3082	3873	3076	4766	4758	4769	4889	3855	4816	4898	4887
3798	100.0												
3082	100.0	100.0											
3873	100.0	100.0	100.0										
3076	100.0	100.0	100.0	100.0									
4766	95.6	95.6	95.6	95.6	95.6								
4758	95.6	95.6	95.6	95.6	95.6	100.0							
4769	95.6	95.6	95.6	95.6	95.6	100.0	100.0						
4889	95.6	95.6	95.6	95.6	95.6	100.0	100.0	100.0					
3855	95.6	95.6	95.6	95.6	95.6	100.0	100.0	100.0	100.0				
4816	95.6	95.6	95.6	95.6	95.6	100.0	100.0	100.0	100.0	100.0			
4898	95.6	95.6	95.6	95.6	95.6	100.0	100.0	100.0	100.0	100.0	100.0		
4887	95.6	95.6	95.6	95.6	95.6	100.0	100.0	100.0	100.0	100.0	100.0	100.0	
3181	95.6	95.6	95.6	95.6	95.6	100.0	100.0	100.0	100.0	100.0	100.0	100.0	100.0

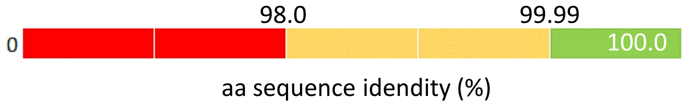

Amino acid (aa) identity of Colicin E5 [%]

n= 2 isolates

***cea*E – Colicin E5 activity gene [UniProtKB: P18000]**

Isolate-ID	REF<br>P18000	Isolate-ID<br>4696
4696	98.63	
4707	98.63	100

***imm* – Colicin E5 immunity gene [UniProtKB: P13476]**

Isolate-ID	REF<br>P13476	Isolate-ID<br>4696
4696	100	
4707	100	100

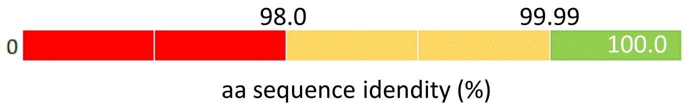

***lys2* – Colicin E5 lysin gene [UniProtKB: P13344]**

Isolate-ID	REF<br>P13344	Isolate-ID<br>4696
4696	89.362	
4707	89.362	100

Amino acid (aa) identity of <u>Colicin E7</u> [%]

n= 5 isolates

ceaG **–** Colicin E7 activity gene [UniProtKB: Q47112]

Isolate-ID	Q47112 (REF)	4696	4707	4769	4766
4696	91.46				
4707	91.46	100.00			
4769	91.46	100.00	100.00		
4766	98.34	100.00	100.00	100.00	
4758	91.46	100.00	100.00	100.00	100.00

ceiG **–** Colicin E7 immunity gene [UniProtKB: Q03708]

Isolate-ID	Q03708 (REF)	4696	4707	4769	4766
4696	67.82				
4707	67.82	100.00			
4769	67.82	100.00	100.00		
4766	67.82	100.00	100.00	100.00	
4758	67.82	100.00	100.00	100.00	100.00

celG **–** Colicin E7 lysin gene [UniProtKB: Q03709]

Isolate-ID	Q03709 (REF)	4696	4707	4769	4766
4696	91.49				
4707	91.49	100.00			
4769	91.49	100.00	100.00		
4766	91.49	100.00	100.00	100.00	
4758	91.49	100.00	100.00	100.00	100.00

0 98.0 99.99 100.0

aa sequence idendity (%)

***mcj*A – Microcin J25 precursor [UniProtKB: Q9X2V7]**

Amino acid (aa) identity of <u>Microcin J25</u>[%]

n= 4 isolates

Isolate-ID	Q9X2V7 (REF)	6215	4107	3363
6215	100			
4107	100	100		
3363	100	100	100	
3333	100	100	100	100

***mcj*B – Microcin J25 processing gene [UniProtKB: Q9X2V8]**

Isolate-ID	Q9X2V8 (REF)	6215	4107	3363
6215	100			
4107	100	100		
3363	100	100	100	
3333	100	100	100	100

***mcjC* – Microcin J25 processing gene [UniProtKB: Q9X2V9]**

Isolate-ID	Q9X2V9 (REF)	6215	4107	3363
6215	100			
4107	100	100		
3363	100	100	100	
3333	100	100	100	100

***mcjD* – Microcin J25 export gene [UniProtKB: Q9X2W0]**

Isolate-ID	Q9X2W0 (REF)	6215	4107	3363
6215	100			
4107	100	100		
3363	100	100	100	
3333	100	100	100	100

0 98.0 99.99 100.0

aa sequence idendity (%)

Amino acid (aa) identity of Microcin H47[%]

n= 3 isolates

***mch*B – Microcin H47 precursor [UniProtKB: P62530]**

Isolate-ID	P62530 (REF)	3798 (Isolate-ID)	3278 (Isolate-ID)
3798	100		
3278	100	100	
3082	100	100	100

***mchC* – Microcin H47 gene [UniProtKB: Q2WEK8]**

Isolate-ID	Q2WEK8 (REF)	3798 (Isolate-ID)	3278 (Isolate-ID)
3798	100		
3278	100	100	
3082	100	100	100

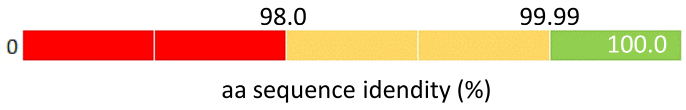

***mchI* – Microcin H47 immunity gene [UniProtKB: O86200]**

Isolate-ID	O86200 (REF)	3798 (Isolate-ID)	3278 (Isolate-ID)
3798	100		
3278	100	100	
3082	100	100	100
